# Pan-genome-based characterization of *SiGRAS* transcription factors and prediction of hub genes correlated with plant height in foxtail millet (*Setaria italica* L.)

**DOI:** 10.1186/s12870-026-09696-z

**Published:** 2026-08-07

**Authors:** Kangni Han, Zhilan Wang, Ziyi Li, Shichao Lian, Hongpo Wu, Xiaofen Du, Yuxin Li, Yanfang Li, Huan Yang, Linyi Zhang, Jun Wang

**Affiliations:** 1 Houji Laboratory; Key Laboratory of Minor Crop Germplasm Innovation and Molecular Breeding (Ministry–Province Co-construction); Shanxi Engineering Research Center for Foxtail Millet Genetic Improvement; Changzhi Key Laboratory of Foxtail Millet Genetic Improvement; Millet Research Institute, Shanxi Agricultural University, Changzhi, 046000 China; 2 College of Agriculture, Shanxi Agricultural University, Taigu, 030800 China

**Keywords:** Foxtail millet, *GRAS* transcription factors, Pan-genome, Structural variation, Plant height

## Abstract

**Background:**

The *GRAS* gene family, a class of plant-specific transcription factors, plays pivotal roles in diverse biological processes, including plant growth, development, and stress responses. Although the *SiGRAS* family has been previously characterized in foxtail millet, those studies were restricted to a single reference genome, overlooking critical genetic variations.

**Results:**

To overcome the inherent limitations of gene family analysis using a single reference, we systematically investigated the *SiGRAS* gene family using pan-genome data generated from 110 foxtail millet accessions together with the *xiaomi* and Yugu1 genomes. Based on a rigorously filtered pan-genome catalog, we identified a total of 949 *SiGRAS* pan-genes, comprising 1 core, 20 near-core, 50 dispensable, and 878 private genes. Phylogenetic analysis classified these genes into 9 distinct subfamilies. Evolutionary analysis based on Ka/Ks ratios revealed that 8 *SiGRAS* genes underwent purifying selection, while *SiGRAS17* and *SiGRAS2* were subjected to positive selection in the majority of accessions. Notably, we observed that structural variations (SVs) significantly influenced the expression levels of *SiGRAS63*, and altered both conserved domains and gene structure in specific accessions. Furthermore, we identified four hub genes correlated with plant height by integrating RNA-seq data with gene co-expression regulatory network.

**Conclusions:**

This study lays a foundation for understanding the diversity of the *SiGRAS* gene family and provides a valuable resource for future function studies of *SiGRAS* genes in foxtail millet.

**Supplementary Information:**

The online version contains supplementary material available at https://doi.org/10.1186/s12870-026-09696-z.

## Background

The *GRAS* gene family is a critical group of plant-specific transcriptional regulators, named after its first identified members: GAI (Gibberellic Acid Insensitive), RGA (Repressor of GA1), and SCR (SCARECROW) [1]. GRAS proteins typically consist of 400–770 amino acids and are characterized by a conserved C-terminal GRAS domain containing five signature motifs: LHRI (Leucine-Heptad Repeat I), VHIID (Val-His-Ile-Ile-Asp), LHRII (Leucine-Heptad Repeat II), PFYRE (Pro-Phe-Tyr-Arg-Glu), and SAW (Ser-Ala-Trp) [1, 2]. The C-terminal motifs are highly conserved, particularly the strictly conserved VHIID, whereas the N-terminal region exhibits significant variability, conferring functional specificity to individual GRAS proteins [2]. In *Arabidopsis thaliana*, the *GRAS* gene family was categorized into 8 distinct subfamilies: *DELLA*, *PAT1* (*PHYTOCHROME A SIGNAL TRANSDUCTION 1*), *HAM* (*HAIRY MERISTEM*), *SHR* (*SHORTROOT*), *SCR*, *LAS* (*LATERAL SUPPRESSOR*), *SCL3* (*SCARECROW-LIKE3*), and *SCL4/7* [3]. Furthermore, subsequent genome-wide studies in cereal crops have revealed significant lineage-specific expansions, with subfamily numbers increasing to 14 in rice [4], 13 in wheat [5], and 13 in foxtail millet [6].

*GRAS* transcription factors are involved in a broad range of biological processes, including developmental regulation, hormone signaling, and adaptation to abiotic stresses. Different *GRAS* subfamilies contribute to these processes in distinct manners. *SHR* and *SCR* subfamilies function coordinately in radial patterning; *SCR* is involved in cell division in the cortex and endodermis of roots in *Arabidopsis*, while *SHR* regulates asymmetric division and determine cortical cell fate by activating *SCR*, thereby driving endodermal differentiation [7–9]. LAS promotes lateral bud or tiller bud formation by regulating lateral meristem development [10, 11], while HAM functions on adjacent tissues in a noncellular autonomous manner to maintain shoot apical meristem activity [12]. DELLA proteins function as central repressors in gibberellin (GA) signaling, and their proteasomal degradation can relieve growth constraints to activate GA-responsive processes, including seed germination and hypocotyl elongation [13, 14]. SCL3 acts as a DELLA antagonist in the GA signaling pathway to maintain a critical balance between plant development and abiotic stress responses [15]. PAT1 functions as a signal transduction intermediate in the phytochrome pathway to regulate photomorphogenesis and modulate responses to multiple abiotic stresses such as salinity, heat, cold, and waterlogging [16]. Similarly, SCL4/7 plays a critical role in adaptation to abiotic stresses; for instance, *PeSCL7* in poplar and *GhSCL4* in cotton, confer drought and salinity tolerance by enhancing antioxidant enzyme activity and maintaining ion homeostasis, respectively [17, 18].

Foxtail millet (*Setaria italica* L.) has emerged as an ideal model system for functional genomics in C4 grass crops [19, 20]. Genome-wide analyses have identified approximately 52 to 60 *SiGRAS* genes characterized by an uneven chromosomal distribution enriching on chromosome 9 but excluding chromosome 6 [6, 21, 22]. Structurally, all members of *SiGRAS* genes possess a conserved C-terminal GRAS domain, and a divergent N-terminal region responsible for functional specificity [3]. Phylogenetic analysis has classified them into 13 subfamilies, namely *LISCL*, *PAT1*, *SHR*, *HAM*, *DELLA*, *SCL3*, *SCL4/7*, *SCR*, *DLT*, *LAS*, *OS19*, *OS4*, and *OS43* [6]. Currently, the tissue-specific expression patterns of these subfamilies have been preliminarily documented. For instance, *LAS* subfamily members, such as *SiGRAS01* and *SiGRAS22*, most *PAT*1 subfamily members, including *SiGRAS04* and *SiGRAS06*, and *HAM* subfamily members, such as *SiGRAS28*, were highly expressed in leaves [6, 22], while *SHR* subfamily members, such as *SiSHR1* and *SiSHR2*, were specifically highly expressed in roots and *DELLA* subfamily members, such as *SiGRAS48*, showed high expression levels in stems [6, 23]. With respect to their biological functions, *DELLA* subfamily members *SiGRAS13* and *SiGRAS25*, key repressors in GA signaling, were significantly upregulated under exogenous paclobutrazol (PAC) treatment, accompanying with reduced endogenous gibberellin levels to regulate plant height [6]. The *SCR* subfamily member *SiGRAS05* was markedly upregulated in roots under PEG stress, suggesting that it may enhance drought resistance through the regulation of root development [22]. Furthermore, *SiGRAS04*, a *PAT1* subfamily member, responded broadly to various treatments, including IAA, GA_3_, MeJA, ABA, PEG, and low temperature (4℃) [22]. These findings provided a foundation for functional genomic studies of *SiGRAS* family and support their potential applications in the genetic improvement of foxtail millet.

To date, all those studies on *SiGRAS* family have predominantly relied on a single reference genome. As a single reference genome represents only one haplotype, it is inherently limited in capturing the full spectrum of presence/absence variations (PAVs), copy number variations (CNVs), and allelic diversity across accessions. Consequently, many lineage-specific genes and key genetic variations of *SiGRAS* may be insufficiently captured and even missed. In contrast, pan-genome-based family analysis offers a crucial strategy to overcome this limitation by consolidating high-quality genome assemblies from numerous accessions. The approach enables systematic identification of gene resources and supports a more thorough investigation of gene function and evolution.

In this study, we performed a comprehensive genome-wide analysis of the *SiGRAS* gene family utilizing a total of 112 foxtail millet accessions, including 110 accessions from the pan-genome [24], *xiaomi* and Yugu1. This study aims to: (i) characterize its evolutionary history and selection pressure; (ii) assess the impact of SVs on expression and conserved domains; and (iii) identify key hub genes associated with plant height through integrating RNA-seq data with gene co-expression network analysis. This study presents the first pan-genome-scale analysis of the *SiGRAS* gene family in foxtail millet, providing a foundation for understanding the diversity, evolution, and potential functions of *SiGRAS* genes.

## Results

### Identification of *SiGRAS* family genes

A total of 5,502 *SiGRAS* genes were identified across 112 foxtail millet accessions using HMMER, with an average of 49.12 genes per accession. Multi-database analysis further confirmed that all identified genes contained the conserved GRAS domain. The number of *SiGRAS* family genes was largely conserved among accessions, as indicated by a coefficient of variation (CV) of 5.32%, although six accessions exhibited extreme variation (|Z-score| > 2.58, *p* < 0.01). Notably, accession Q13 harbored 60 *SiGRAS* genes, representing a significant 22.1% expansion over the population average (Z-score = 4.16, *p* < 0.001) (Additional file 1: Table S1).

We further found that there were no statistically significant differences in *SiGRAS* gene number among wild, landrace, and cultivar groups (*p* = 0.13). However, wild accessions exhibited the highest mean number (49.71 ± 2.7), followed by cultivars (49.25 ± 2.5) and landraces (48.51 ± 2.6) (Additional file 1: Table S2). Meanwhile, the wild group showed the highest CV (5.379%), reflecting greater genetic diversity within natural populations. In contrast, cultivars displayed the lowest CV (5.030%), consistent with the domestication bottleneck effect, suggesting a reduction in genetic diversity due to intensive breeding selection. Additionally, landraces exhibited the highest proportion of accessions with significant variations based on Z-scores (4/41, 9.8%), followed by cultivars (3/36, 8.3%) and wild accessions (2/35, 5.7%) (Additional file 1: Table S3).

### PAV analysis of *SiGRAS* gene family

**Fig. 1:**
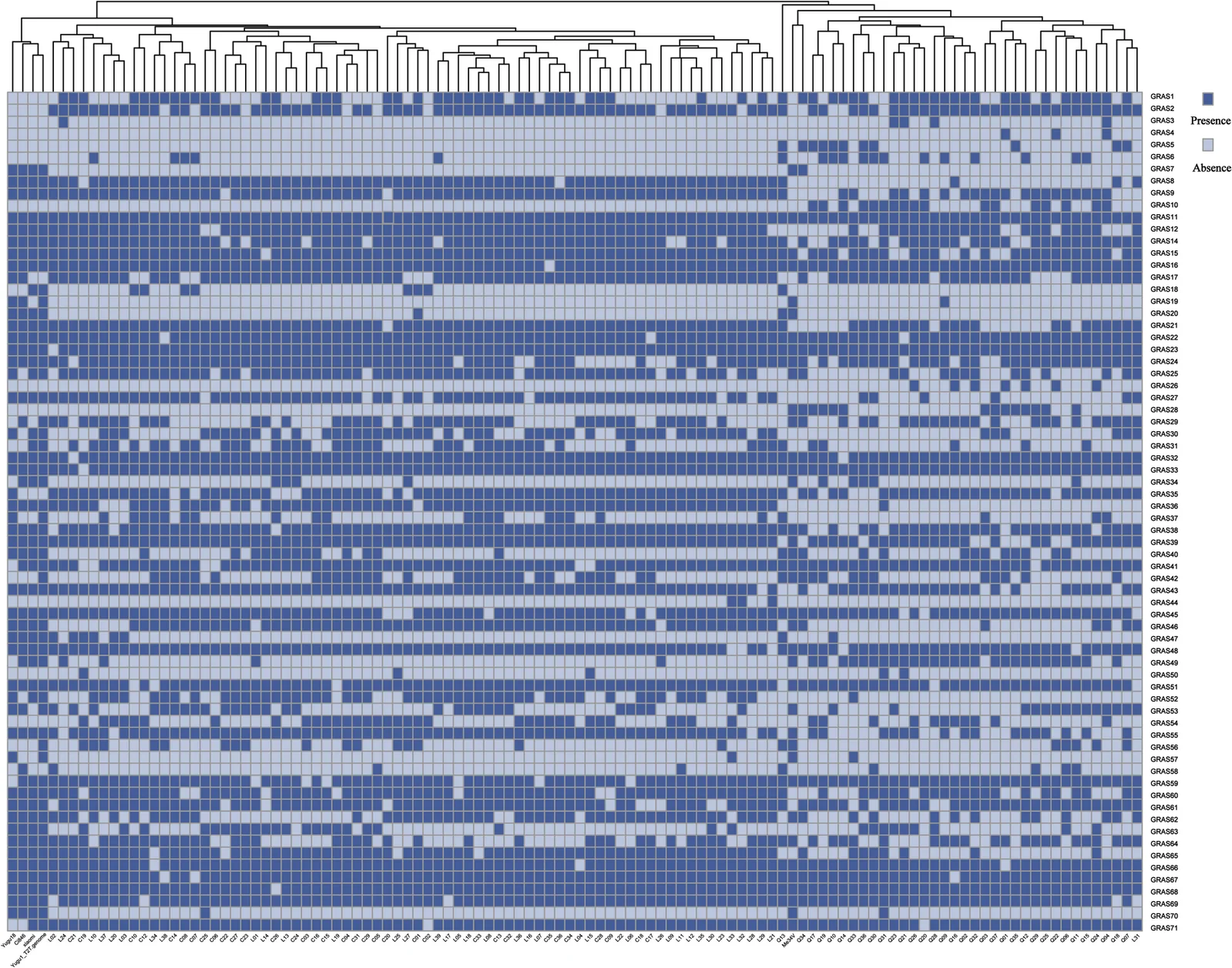
Heatmap of the presence and absence of *SiGRAS* genes, excluding private genes, across 112 foxtail millet accessions. Column represents each accession, and row represents each *SiGRAS* gene. Dark blue indicates presence, whereas light blue absence

A non-redundant dataset including 949 high-confidence *SiGRAS* pan-genes was identified through PAV analysis by integrating the pan-gene catalog, 5,502 initial *SiGRAS* genes and private genes. Among these, there were one core gene (0.10%), 20 near-core genes (2.11%), 50 dispensable genes (5.27%), and 878 private genes (92.52%). The high proportion of private genes highlighted an extensive accession-specific diversification within the *SiGRAS* family, likely driven by local adaptation pressures (Additional file 2: Fig. S1). In contrast, the single core gene, *SiGRAS11*, was uniformly present across all accessions, indicating its biological function conservation throughout domestication (Fig. 1; Additional file 2: Fig. S1). Hierarchical clustering based on gene PAV profiles separated these accessions into two major clades, and wild accessions clearly segregated from landrace and cultivated accessions (Fig. 1), indicating distinct evolutionary trajectories and providing an evidence for domestication signatures within the *SiGRAS* gene family.

### Phylogenetic analysis

**Fig. 2:**
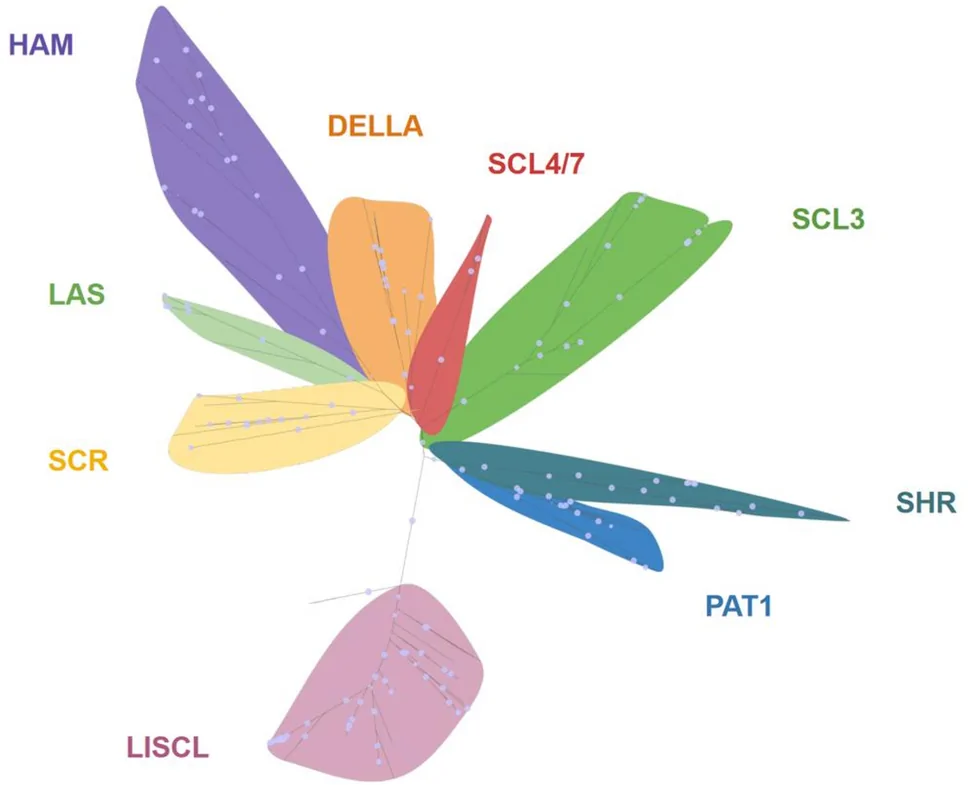
Phylogenetic tree of *GRAS* genes in foxtail millet and *Arabidopsis*. Different colors represent distinct subfamilies. Bootstrap support values (≥ 70%) are indicated at the nodes

Rooted with *Arabidopsis GRAS* genes, phylogenetic analysis was conducted by the Maximum Likelihood (PAML), and 949 *SiGRAS* pan-genes were classified into 9 distinct clades: *LISCL*, *HAM*, *PAT1*, *SHR*, *SCL3*, *SCR*, *LAS*, *SCL4/7*, and *DELLA* (Fig. 2). The *LISCL* clade was the largest, containing 365 members, indicating a significant expansion during evolution, while *SCL4/7* was the smallest, containing 23 members. Notably, *AtGRAS* genes were distributed across all clades, suggesting that the divergence of *GRAS* genes predated the speciation of foxtail millet and *Arabidopsis*, which was consistent with prior studies [3, 6].

### *SiGRAS* genes are subjected to different selection pressures

**Fig. 3:**
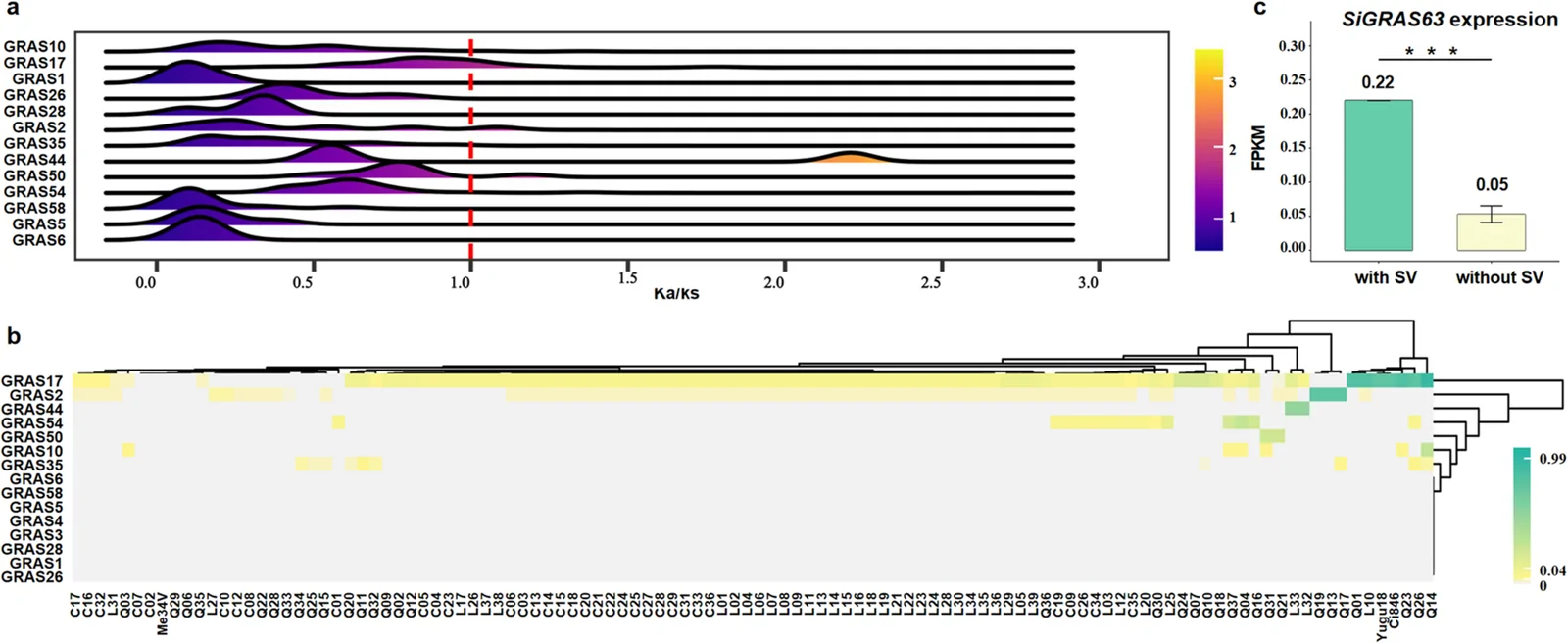
Ka/Ks value of *SiGRAS* genes and effect of SVs on genes. **a** Distribution of Ka/Ks value of *SiGRAS* genes in 112 accessions. **b** Heatmap of the occurrence frequency of each *SiGRAS* gene with a Ka/Ks ratio > 1 among different accessions. **c** The expression of *SiGRAS63* was significantly affected by SVs. With SV group: *n* = 3 (one accession, three replicates); Without SV group: *n* = 15 (five accessions, three replicates). *, **, *** indicate *P* < 0.05, *P* < 0.01, and *P* < 0.001, respectively

To investigate the evolutionary dynamics of the *SiGRAS* family, we evaluated the selection pressure by calculating the Ka/Ks ratios for orthologous pairs across 112 accessions based on Yugu1 genome (Yugu1_T2T.genome) (Fig. 3a and b). A total of 13 *SiGRAS* genes yielded reliable Ka/Ks ratios, while the others were excluded due to the reference ortholog lack or poor alignment quality. For these 13 genes, the peaks of the Ka/Ks ratio distributions were all below 1, as shown in the ridgeline plot (Fig. 3a), suggesting that purifying selection may have predominated during evolution. In particular, *SiGRAS44* exhibited a secondary peak between 2.0 and 2.5, suggesting that it may have experienced positive selection in certain accessions. Furthermore, two genes, *SiGRAS17* and *SiGRAS2*, exhibited a Ka/Ks value exceeding 1 in the majority of accessions (Fig. 3b), implying that they may have been subject to positive selection during domestication.

### SVs impact the expression and structure of *SiGRAS* genes

**Fig. 4:**
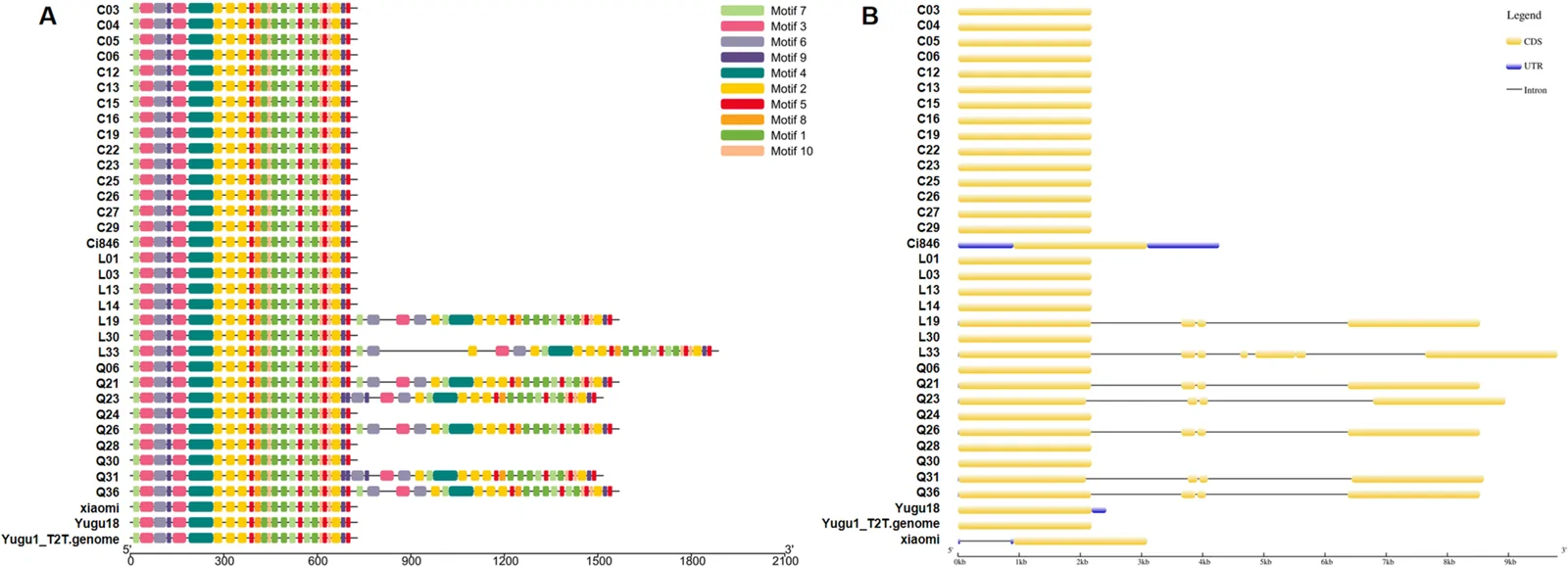
Conserved domains (**a**) and gene structure (**b**) of *SiGRAS63* in 35 accessions

**Fig. 5:**
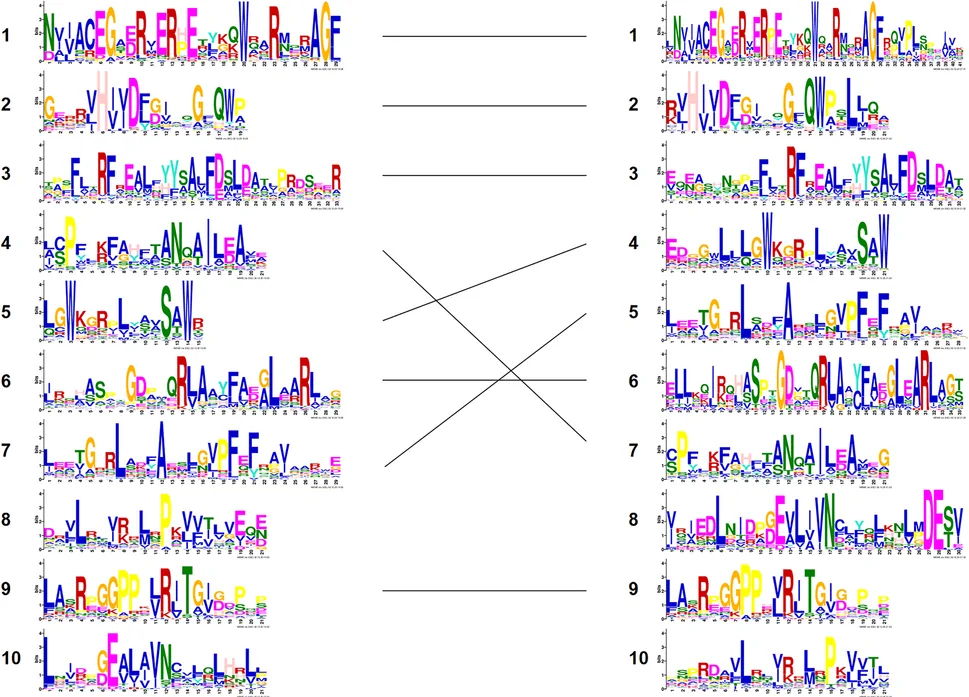
Motif correspondence between reference SiGRAS (left) and L30 (right). Motif is numbered independently by decreased E-value. Line represents matching motifs between two accessions, indicating conserved domains; unmatched motifs suggest potential alteration by SVs

A total of 56 *SiGRAS* genes were identified in the Yugu1_T2T.genome; based on those, 214 SVs were identified within genic regions and 2-kb flanking regions of these genes across 110 accessions and *xiaomi*. These SVs were annotated as upstream, UTR5, exon, intron, UTR3, downstream, and intergenic (Additional file 1: Table S4). To evaluate whether SVs modulate the transcriptional activity of *SiGRAS* genes, we compared the expression levels between accessions “with SVs” and “without SVs” for each gene. Wilcoxon rank-sum test and Student’s t-test analysis revealed that only one gene, *SiGRAS63* (*Seita.9G174900* in the Yugu1_T2T.genome), present in 35 accessions, exhibited an extremely significant difference in expression levels (Fig. 3c), indicating that the presence of SVs markedly influenced its expression. Furthermore, we found that the motif composition and arrangement of conserved domains of this gene remained highly consistent across most accessions. However, notable SVs were observed in seven accessions (L19, L33, Q21, Q23, Q26, Q31, and Q36) (Fig. 4a); correspondingly, the gene displayed multi-exon structures in these accessions (Fig. 4b), but a simple single-exon structure in the others. Furthermore, accession L30, with the largest number of SV-affected *SiGRAS* genes, was selected as a representative accession to investigate conserved domains variants, and two motifs, Motifs 8 and 10, were found to be absent in L30 (Fig. 5).

### Identification of hub *SiGRAS* genes regulating plant height via transcriptome and co-expression network analysis

**Fig. 6:**
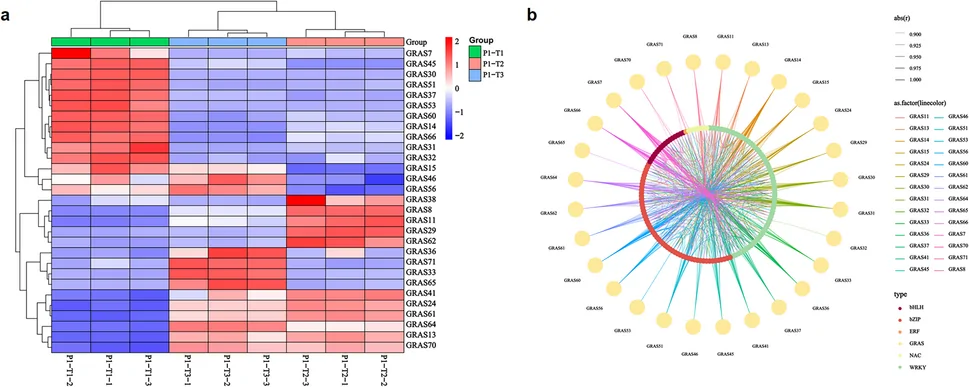
Expression patterns (**a**) and co-expression network analysis (**b**) of 29 *SiGRAS* DEGs across three developmental stages of plant height. (**a**) Heatmap showing expression levels of 29 *SiGRAS* DEGs across three plant height developmental stages (T_1_, T_2_, T_3_) of accession C24 (P_1_). (**b**) Co-expression network between 29 *SiGRAS* DEGs and TFs. Yellow outer circle node represents *SiGRAS* DEGs, and inner circle node represents TFs colored by family group. Edge indicates significant pearson correlations (|r| ≥ 0.9, *p* < 0.01), with edge thickness proportional to correlation strength. Edge color corresponds to specific *SiGRAS* DEGs. Candidate hub genes were identified based on a degree threshold of ≥ 40

Furthermore, we analyzed hub genes regulating plant height by integrating the differentially expressed genes (DEGs) from three developmental stages of plant height [25] in accession C24 with gene co-expression regulatory network. In total, we identified 29 *SiGRAS* genes exhibiting differential expression (Additional file 1: Table S5). Hierarchical clustering of expression patterns of these genes (Fig. 6a) revealed that 12 *SiGRAS* genes, consisting of *SiGRAS7*, *SiGRAS45*, *SiGRAS30*, *SiGRAS51*, *SiGRAS37*, *SiGRAS53*, *SiGRAS60*, *SiGRAS14*, *SiGRAS66*, *SiGRAS31*, *SiGRAS32*, and *SiGRAS15*, showed significantly higher expression during the jointing stage (T_1_), followed by progressive down-regulation during the heading (T_2_) and harvest (T_3_) stages; whereas 11 *SiGRAS* genes, including *SiGRAS38*, *SiGRAS8*, *SiGRAS11*, *SiGRAS29*, *SiGRAS62*, *SiGRAS41*, *SiGRAS24*, *SiGRAS61*, *SiGRAS64*, *SiGRAS13*, and *SiGRAS70*, exhibited low initial expression during T_1_ but were progressively up-regulated during the T_2_ and T_3_ stage; the others displayed an initial increase followed by a decrease, with peak expression at T_2_. Subsequently, to identify key regulatory nodes, we constructed a gene co-expression regulatory network by integrating these *SiGRAS* DEGs with 6 major transcription factor (TF) families (*bHLH*, *bZIP*, *ERF*, *GRAS*, *NAC*, and *WRKY*) acting as potential regulators. The result revealed that four genes, namely *SiGRAS7*, *SiGRAS66*,* SiGRAS14*, and *SiGRAS31*, corresponding to *Seita.1G048800*,* Seita.9G355300*, *Seita.2G372300*, and *Seita.4G219200* in the Yugu1_T2T.genome, respectively, exhibited the highest degree values (46, 42, 41, and 41, respectively) (Fig. 6b; Additional file 1: Table S6 and Table S7), corresponding to the extreme upper tail of the degree distribution (top 2.5%). Therefore, these genes were considered as core hub genes and key candidate regulators for plant height. Notably, function annotation of these candidate genes indicated that they may act as key developmental regulators: *SiGRAS66* and *SiGRAS14* are predicted to encode SHORT-ROOT 1 proteins, while *SiGRAS31 and SiGRAS7* are predicted to encode an ortholog of MONOCULM 1 (Additional file 1: Table S5).

## Discussion

In this study, we constructed a high-confidence pan-genome catalog of *SiGRAS* gene family based on stringent dual criteria of sequence similarity and chromosomal synteny, and then defined 949 high-confidence *SiGRAS* pan-genes across 112 foxtail millet accessions, including 1 core gene, 20 near-core genes, 50 dispensable genes, and 878 private genes (Additional file 2: Fig. S1). Notably, the proportion of private *SiGRAS* genes (92.5%) was substantially higher than that reported in other transcription factor families, such as maize *TPS* (3%) and barley *bHLH* (10%) [26, 27]. The high proportion of private genes identified in this study may be attributable to the larger number of accessions examined and the stringent synteny-based orthology criteria applied, which together increased the likelihood of detecting accession-specific genes. Additionally, in our pan-genome dataset, most genome assemblies were at the scaffold level and some remained at the contig level (Additional file 1: Table S8), which may also have inflated the apparent number of private genes. Collectively, these findings highlight the power of large-scale pan-genome analysis to uncover hidden genetic diversity.

Furthermore, phylogenetic analysis classified 949 *SiGRAS* pan-genes into 9 subfamilies: *LISCL*, *HAM*, *PAT1*, *SHR*, *SCL3*, *SCR*, *LAS*, *SCL4/7* and *DELLA*, different from those (from 10 to 13 subfamilies) in previous studies [6, 21, 22]. This difference was likely attributable to three methodological factors: one outgroup (*Arabidopsis*), a higher bootstrap threshold (≥ 70%) and a stringent pan-genome non-redundancy filtering, which may have limited the resolution of grass-specific subfamilies and lead to a more conservative classification. Additionally, the subfamilies *DLT*, *OS19*, *OS4*, and *OS43* reported by Fan et al. (2021) were absent from our classification, which was likely because of non clear orthologs of these subfamilies in *Arabidopsis.*

Our study revealed that the *SiGRAS* family underwent dynamic changes during domestication. Notably, the number of *SiGRAS* genes showed a stepwise decline: wild accessions > landraces > cultivated varieties, consistent with the domestication trajectory of foxtail millet [28]. Despite mean gene numbers differed across groups, CV remained remarkably stable (5.03%-5.38%) (Additional file 1: Table S2), indicating that variation level within the *SiGRAS* family was largely conserved. Interestingly, landraces and wild accessions exhibited the same CV (5.38%), suggesting that traditional landrace farming systems may have maintained genetic diversity as effectively as natural ecosystems. In contrast, the lower genetic variation observed in modern cultivars (CV = 5.03%) may reflect the more rapid fixation of favorable alleles during breeding [28, 29]. Collectivity, the stepwise decline in CV across three groups reflected a typical domestication bottleneck and was also consistent with the purifying selection identified by the Ka/Ks analysis. Furthmore, only 13 genes yielded reliable Ka/Ks values in 949 *SiGRAS* pan-genes, which was largely due to the high proportion of private genes (92.5%), and the exclusion of multi-copy or poorly aligned sequences (coverage < 70% or identity < 70%). Among 13 *SiGRAS* genes, 3 genes, *SiGRAS44*, *SiGRAS17* and *SiGRAS2*, were found to experience postive selection (Ka/Ks > 1), but further confirmation using PAML was required. Specifically, *SiGRAS44*, a putative ortholog of rice *OsNSP2* involved in nodulation signaling and development, was detected only in three landraces (L21, L32, L33), reflecting its specific local adaptation. *SiGRAS17* and *SiGRAS2*, putative orthologs of rice *OsCIGR2* and *OsMOC1*, respcectively, involved in stress responses and gibberellin signaling, were widely detected across almost all accessions (95 and 94 accessions, respectively). Their broad distribution implied that these positively selected genes may have retained conserved functions during domestication, although further experimental validation was needed in foxtail millet. Moreover, we found that *SiGRAS63* showed a significant expression difference between accessions with and without SVs (Fig. 3c), and also displayed corresponding changes in gene structure and conserved domains (Fig. 4), suggesting that it may be involved in SV-mediated functional variation associated with domestication, although further validation was still required.

We also investigated 29 *SiGRAS* DEGs significantly associated with plant height based on RNA-seq and co-expression network analyses across three developmental stages. Functional annotation revealed that these genes were exclusively enriched in plant hormone signaling pathways (Additional file 1: Table S5). Notably, 12 genes reached their highest expression levels at the jointing stage (T_1_), while 11 genes exhibited continuous up-regulation throughout the developmental stages. Finally, four hub genes, *SiGRAS7*, *SiGRAS14*, *SiGRAS31*, and *SiGRAS66*, were identified through co-expression network analysis. Among them, *SiGRAS14* and *SiGRAS66* were orthologs of *SHORT-ROOT* (*SHR*), which regulated radial patterning and vascular development in *Arabidopsis* [7, 30] and controlled plant height, tiller number, and yield in rice [31]. Additionally, *SiGRAS7* and *SiGRAS31* were orthologs of rice *MONOCULM* 1 (*MOC1*), a key regulator of axillary meristem initiation, tillering and plant architecture [32]. These findings strongly implyed that these hub genes may be key candidates involved in plant height development in foxtail millet. Therefore, they could serve as potential targets for molecular breeding to optimize plant architecture, however, further experimental validation, such as expression analysis across multiple accessions and genetic transformation analysis, were required to confirm their roles in plant height development.

## Conclusion

In this study, we comprehensively characterized the *SiGRAS* family using a pan-genome dataset comprising 110 accessions, along with the Yugu1 reference genome and *xiaomi* genome. Through systematic analysis, 949 high-confidence *SiGRAS* pan-genes were identified, including numerous private genes, together with 1 core gene, 20 near-core genes, and 50 dispensable genes, of which 13 were predominantly subjected to purifying selection, whereas 3 genes showed positive selection, and especially *SiGRAS17* and *SiGRAS2* experienced strong positive selection during domestication. Importantly, we found that SVs affected the gene structure, conserved domains, and expression of *SiGRAS* genes. Furthermore, we identified four candidate hub genes associated with plant height by integrating RNA-seq and co-expression network analysis. Collectively, these findings provided a valuable foundation for future function analysis of *SiGRAS* genes in foxtail millet.

## Materials and methods

### Construction of the non-redundant pan-gene catalog

Genomic sequences and gene annotation files for 112 foxtail millet accessions were obtained from *Setaria*-DB (http://111.203.21.71:8000/download.html) [33]. The Yugu1 genome (Yugu1_T2T.genome) was used as the initial reference template. The longest isoform for each gene was retained for furtheeer analysis. Pairwise synteny analysis was conducted using jcvi.compara.catalog ortholog with --cscore = 0.8 as the synteny score threshold. Genes showing syntenic matches between the initial reference template and other accessions were regarded as syntenic orthologs, whereas genes lacking syntenic matches were considered absent and incorporated into the pan-gene dataset. The preliminary pan-gene dataset was then self-compared using the same JCVI synteny workflow with a stringent cutoff of --cscore = 0.95, and all of syntenic genes were collapsed to retain one representative gene per group.

### Identification of *SiGRAS* gene family

The Hidden Markov Model (HMM) profile for the GRAS domain (PF03514) was downloaded from the Pfam database via InterPro (https://www.ebi.ac.uk/interpro). *SiGRAS* candidates were identified by screening protein sequences of each accession against the HMM profile using HMMER 3.3.2, with an E-value cutoff of 1 × 10^− 5^ and a minimum domain coverage of 90% of the HMM model length. Conserved domain of protein sequence was validated using three databases: NCBI (https://www.ncbi.nlm.nih.gov/Structure/bwrpsb/bwrpsb.cgi), SMART (https://smart.embl.de/smart/batch.pl), and InterPro. Proteins lacking a complete GRAS domain were excluded from further analysis.

### PAV analysis of *SiGRAS* gene family

To characterize the PAV of the *SiGRAS* gene family, reliable *SiGRAS* family members were subsequently retrieved from the non-redundant pan-gene catalog based on protein sequences and conserved domain sequences. Private genes excluded in the pan-gene catalog were identified through HMM-based domain screening and then added to the final *SiGRAS* pan-gene dataset. Subsequently, a binary PAV matrix was generated, where “1” and “0” represented the presence and absence of *SiGRAS* genes in accessions, respectively. Finally, hierarchical clustering was performed based on this matrix and the results were visualized as a heatmap using the ComplexHeatmap package (v2.14.0) in R 4.0.3 [34, 35]. The overall workflow for obtaining the high-confidence *SiGRAS* pan-genes was shown in Additional file 3: Fig. S2.

### Phylogenetic analysis of *SiGRAS* gene family

The conserved domain sequences were isolated from *Arabidopsis* and foxtail millet (Phytozome v13) using HMMER with an E-value cutoff of < 1e^− 5^ and coverage > 90% of the HMM length, and then used for phylogenetic analysis. Multiple sequence alignment was performed using MUSCLE v3.8.31 [36]. The resulting alignment was further refined by trimming columns with a gap threshold of ≥ 0.8 and a conservation threshold of ≤ 80% using TrimAl v1.4.1 [37]. Phylogenetic tree was constructed using the Maximum Likelihood method implemented in IQ-TREE 2 (v2.2.0) [38]. The best‑fit substitution model was automatically selected using ModelFinder with the -m MFP option. Branch support was evaluated using ultrafast bootstrap with 1,000 replicates (-B 1000) and the --bnni option to reduce model violations. Phylogenetic tree was visualized in iTOL v6 (https://itol.embl.de/) [39], where branches with bootstrap support values ≥ 70% were displayed.

### Ka/Ks calculation

Orthologous gene pairs within each pan-gene ID were aligned using ParaAT [40], and the orthologous pairs with alignment coverage ≥ 70% and nucleotide identity ≥ 70% were retained for further Ka/Ks calculation. Multi-copy genes were excluded. Ka/Ks ratios were calculated using the Yang-Nielsen codon model in KaKs_Calculator v3.0 [41]. Ridgeline plots of Ka/Ks density distributions were conducted using the ggridges [42] and ggplot2 packages [43] in R v4.0.3 [34]. Heatmaps depicting positive selection frequency (Ka/Ks > 1) were generated with the ComplexHeatmap package (v2.14.0) in R 4.0.3 [34].

### SV annotation and expression analysis of *SiGRAS* genes

SV calls were focused on variants located within genic regions and their 2-kb flanking regions, and those corresponding to the 56 *SiGRAS* genes identified in the Yugu1_T2T.genome were merged into a unified multi-sample VCF file using BCFtools v1.15 [44]. SVs was then annotated using ANNOVAR [45]. The expression data of 56 *SiGRAS* genes across 26 accessions were downloaded from *Setaria*-DB (http://111.203.21.71:8000/multi-omics/transcriptome.html). To evaluate the impact of SVs on gene expression, firstly, genes in group with fewer than 3 samples were excluded, and then the significance testing of gene expression levels between accessions “with SVs” and “without SVs” was conducted using both the Wilcoxon rank-sum test and Student’s t-test.

### Impact of SVs on conserved domains and gene structure

To evaluate the impact of SVs on conserved domains and gene structure of the *SiGRAS* gene family, the conserved domains and gene structures of significantly differentially expressed *SiGRAS* genes were analyzed across 112 accessions. Conserved domains were analyzed using MEME Suite v5.4.1 (https://meme-suite.org/meme/tools/meme) [46]. Gene structures were visualized using GSDS 2.0 (https://gsds.gao-lab.org/) [47]. The accession harboring the largest number of SV-affected *SiGRAS* genes was considered as representative accession. Conserved domain of all the homologous genes in the representative accession was selected to compare with that of the Yugu1_T2T.genome to evaluate the effects of SVs on domain variation. Comparative motif analysis was performed using the MEME algorithm.

### RNA-seq data analysis and gene co-expression network analysis

To investigate the expression dynamics of *SiGRAS* genes, we re-analyzed RNA-seq data (https://www.ncbi.nlm.nih.gov/, Accession: PRJNA1100772) from the accession C24 by Han et al. [25]. Raw reads were applied to quality assessment using FastQC [48], and trimmed with Trimmomatic [37] to remove adapters and low-quality sequences. Clean reads were then aligned to the Yugu1_T2T.genome using HISAT2 [49]. Gene-level raw read counts were generated using featureCounts [50] based on the corresponding genome annotation. Differential expression analysis was conducted using DESeq2 [51] based on raw count data, and genes with a false discovery rate (FDR) < 0.01 and ∣log_2_ FC∣ ≥ 1 were considered differentially expressed. Gene expression levels were normalized as fragments per kilobase of transcript per million mapped reads (FPKM), and DEGs of *SiGRAS* family members were extracted for hierarchical clustering using the R pheatmap package [52]. Gene co-expression network was constructed based on the FPKM values of *SiGRAS* DEGs and core TF families (*bHLH*, *bZIP*, *ERF*, *GRAS*, *NA*C, and *WRKY*) to identify key regulatory nodes controlling plant height. Pearson correlation coefficients between *SiGRAS* DEGs and TF family genes were calculated using R 4.0.3 [34], and gene pairs with p-value < 0.01 and |r| ≥ 0.9 were retained to construct the co-expression network. The co-expression network was visualized using Cytoscape [53], where node connectivity (degree) was calculated using R 4.0.3, and genes with degree ≥ 40 were defined as hub genes.

## Supplementary Information

Additional file 1: Table S1. Evaluation of SiGRAS gene number variations among accessions. Table S2. Descriptive statistics and variance analysis of SiGRAS gene numbers among wild, landrace, and cultivar groups. Table S3. Evaluation of SiGRAS gene number variations among wild, landrace, and cultivar accessions. Table S4. Structural variation-annotated genes of SiGRAS genes in the Yugu1_T2T.genome. Table S5. Identifiers and comprehensive functional annotations of 29 SiGRAS DEGs. Table S6. Edgelist of the SiGRAS genes-transcription factor interaction network. Table S7. Nodelist and topological properties of the SiGRAS gene–transcription factor interaction network. Table S8. Genome assembly and quality evaluation of 112 foxtail millet accessions.

Additional file 2: Fig. S1. Heatmap of presence and absence of SiGRAS genes across 112 foxtail millet accessions.

Additional file 3: Fig. S2. Workflow for constructing the 949 non-redundant SiGRAS pan-gene set.

## Data Availability

All datasets supporting the findings of this study are available within the article and its supplementary materials. RNA-seq data for accession C24 at three key developmental of plant height, which were re-analyzed in this study, are publicly available in the NCBI Sequence Read Archive under BioProject accession PRJNA1100772.
