# Supplementary material for: Pan-genome-based characterization of SiGRAS transcription factors and prediction of hub genes correlated with plant height in foxtail millet (Setaria italica L.)
Source: BMC Plant Biol. 2026 Aug 7;26:1883. doi: 10.1186/s12870-026-09696-z (PMC13733028; doi:10.1186/s12870-026-09696-z)
Supplement: Supplementary file 1 [file 12870_2026_9696_MOESM2_ESM.pdf]

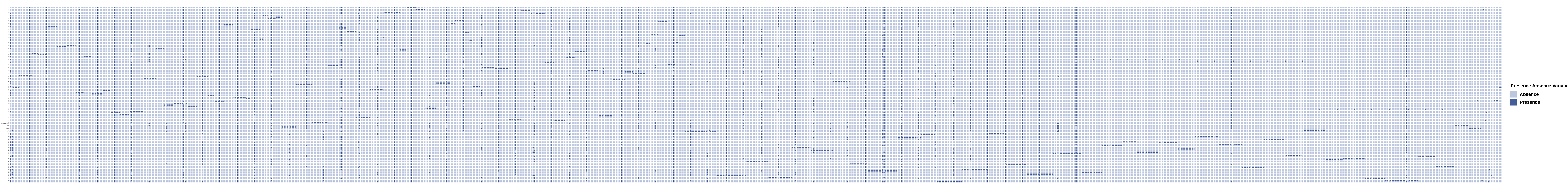

Fig. S1 Heatmap of the presence and absence of *SiGRAS* genes across 112 foxtail millet accessions.
